# Outcomes and Prognostic Factors Following Pars Plana Vitrectomy for Intraocular Foreign Bodies—11-Year Retrospective Analysis in a Tertiary Care Center

**DOI:** 10.3390/jcm11154482

**Published:** 2022-08-01

**Authors:** Mădălina Claudia Hapca, George Adrian Muntean, Iulia Andrada Nemeș Drăgan, Ștefan Cristian Vesa, Simona Delia Nicoară

**Affiliations:** 1 Doctoral School of Medicine, “Iuliu Hațieganu” University of Medicine and Pharmacy, 8, V. Babeș Str., 400012 Cluj-Napoca, Romania; georgemuntean99@gmail.com; 2 Department of Ophthalmology, “Iuliu Hațieganu” University of Medicine and Pharmacy, 8, V. Babeș Str., 400012 Cluj-Napoca, Romania; d_iulia_a@yahoo.com; 3 Ophthalmology Clinic, Emergency County Hospital, 3–5 Clinicilor Str., 400006 Cluj-Napoca, Romania; 4 Department of Pharmacology, Toxicology and Clinical Pharmacology, “Iuliu Hațieganu” University of Medicine and Pharmacy, 400012 Cluj-Napoca, Romania; stefanvesa@gmail.com

**Keywords:** eye injuries, intraocular foreign body, pars plana vitrectomy, retinal detachment, risk factors

## Abstract

Aim: To evaluate the visual outcome of penetrating ocular injuries with a retained intraocular foreign body (IOFB) managed by pars plana vitrectomy (PPV) and to describe the risk factors associated with poor visual acuity and retinal detachment (RD) development. Methods: Medical records of 56 patients with IOFB that were removed by PPV over a period of 11 years (1 January 2010–31 December 2020) were reviewed. We extracted the demographic data, initial and final best corrected visual acuity (BCVA) using standard Snellen chart, IOFB characteristics, complications and surgical details. Outcome was evaluated according to the final BCVA: poor <0.1, good 0.1–<0.5 or excellent ≥0.5. Results: The mean age was 36.1 ± 14.1 (range, 16–71) years and the majority of patients were males (55 out of 56, 98.2%). IOFB was retinal in 27 (48.2%) cases and intravitreal in 29 cases (51.8%). IOFB size was ≤3mm in 26 (46.4%) cases and >3mm in 30 (53.6%) cases. Preoperative RD was identified in 12 (21.4%) cases and endophthalmitis in 17 cases (30.4%). IOFBs larger than 3 mm and retinal location were associated with RD development. Poor visual outcome was associated with initial BCVA, retinal location, RD and endophthalmitis. Conclusion: Initial BCVA, retinal foreign body, RD and endophthalmitis were risk factors for poor visual outcome.

## 1. Introduction

Intraocular foreign bodies (IOFBs) represent a major cause of vision loss, particularly in the working-age population [1,2]. They account for 18–41% of all open globe injuries, thus representing a public health concern worldwide [3,4,5,6]. Open globe injuries with retained IOFBs offer one of the most challenging scenarios to the ophthalmic surgeon due to their complex presentation. Previous studies have shown that the visual outcome in this type of ocular trauma is directly correlated with some predictive factors such as age, initial best corrected visual acuity (BCVA), wound length, IOFB size and the occurrence of complications such as retinal detachment (RD) and endophthalmitis [1,7,8,9,10,11,12,13,14,15,16,17,18,19,20]. In this retrospective case series, we evaluated 56 patients with IOFBs who underwent pars plana vitrectomy (PPV) over an 11-year period, with the aim to outline the prognostic factors with an impact on the visual outcome. We also aim to describe the risk factors associated with poor visual acuity and RD development.

## 2. Materials and Methods

### 2.1. Study Design and Subjects

This retrospective study was carried out in the Department of Ophthalmology, “Iuliu Hațieganu”, University of Medicine and Pharmacy, Emergency County Hospital from Cluj-Napoca, Romania, between 1 January 2010 and 31 December 2020. The study was conducted in accordance with the Declaration of Helsinki and was approved by The Ethics Committees belonging to the “Iuliu Hațieganu” University of Medicine and Pharmacy and the Emergency County Hospital Cluj-Napoca and all the patients signed the informed consent.

The inclusion criterion was represented by IOFB that was extracted by PPV. During the abovementioned 11-year period, 56 cases fell into this category. Both primary and additional surgery procedures (if needed) were performed by the same surgeon (S.D.N.). We reviewed the medical records of the 56 patients and collected data including age, sex, rural or urban setting, type of accident, initial and final BCVA (Snellen Chart), initial ocular findings, characteristics of the IOFB, the surgeries performed and complications. Information on the location, material and size of the IOFB were collected. In perforating cases where IOFB could not be retrieved, the size was noted according to the measurements provided by computer tomography. The location of the entry lesion was classified according to the Ocular Trauma Classification Group [21] and divided into three zones: zone I included the cornea and the limbus; zone II, the 5 mm of sclera immediately posterior to the limbus; and zone III, the sclera at more than 5 mm posterior to the limbus. 

The diagnosis of IOFB and associated lesions has been established by clinical examination, B-scan ultrasonography, computer tomography or orbital radiography. Post-traumatic endophthalmitis was diagnosed based on past ocular trauma history, clinical findings and symptoms at presentation. During surgery, no vitreous tap for culture was taken due to economic reasons. Nevertheless, even with negative cultures, we cannot exclude a diagnosis of endophthalmitis in the presence of clinical signs. The vitreous tap would have identified the specific germ involved, but the antibiotics that can be administered intravitreally are limited. 

### 2.2. Treatment

The goal of treatment was to remove the IOFB and restore the integrity and function of the eye. Primary suture was carried out in patients with leaking wounds. Three-port PPV was performed in all cases. Following core vitrectomy, any adherence between the IOFB and the vitreous or the retina was eliminated, and the IOFB was released into the vitreous cavity and then removed through one enlarged sclerotomy with the intraocular magnet. Additional surgical gestures were dictated by the specific situation: lensectomy/phacoemulsification, if traumatic cataract was associated; endolaser photocoagulation, if retinal lesions were identified. If RD was present, fluid/air exchange with repair of the lesion and silicon tamponade was performed, and in cases with endophthalmitis, intravitreal vancomycin 1 mg/0.1 mL, ceftazidime 2.2 mg/0.1 mL, amikacin 0.4 mg/0.1 mL and dexamethasone 0.4 mg/0.1 mL were injected. 

### 2.3. Statistical Analysis

Statistical analyses were performed using the SPSS Statistics version 20 (SPSS Inc., Chicago, IL, USA). All numerical data are expressed either as the median (minimum–maximum) or as the mean ± standard deviation. All categorical variables are expressed as the number and percentage (*n*, %). Outcome was evaluated according to the final BCVA: poor <0.1, good [0.1–0.5) or excellent ≥0.5. A chi-squared test or Fisher test for categorical variables was applied to compare the differences among subjects. Univariate logistics regression was applied to examine the associations between risk factors and final BCVA. A *p*-value of less than 0.05 was considered statistically significant throughout the study. In case of unequal data distribution, the *p*-value was obtained by the Kruskal–Wallis test.

## 3. Results

### 3.1. Demographic Results

Fifty-six eyes of patients diagnosed with IOFB extracted by PPV were included in this study. The mean age was 36.1 ± 14.1 (range, 16–71) years. The demographic characteristics of the study population are summarized in Table 1. Most of the patients were males, 55 (98.2%), and there was 1 (1.8%) female. Among these, 25 patients (44.6%) had trauma to the right eye and 31 (55.4%) to the left eye; none of them were wearing eye protection at the time of injury. Most accidents happened during household activities and only four (7.1%) at the workplace, being largely caused by hammering metal or during grass cutting. There was no statistically significant correlation between the demographic data and final BCVA scores. 

### 3.2. IOFB Characteristics

**Figure 1:**
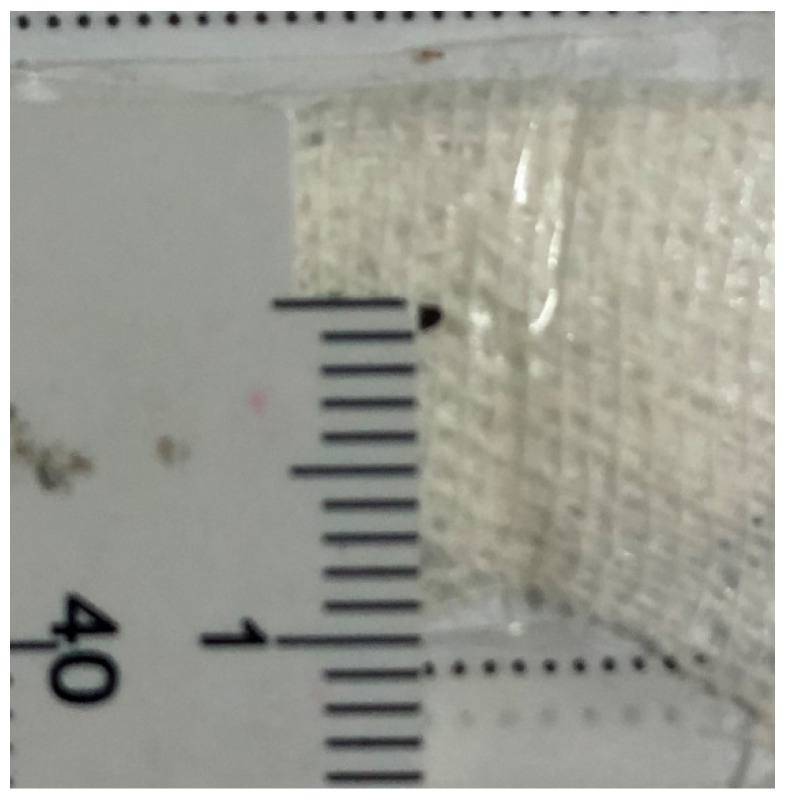
Small IOFB (1 mm). IOFB=Intraocular foreign body.

**Figure 2:**
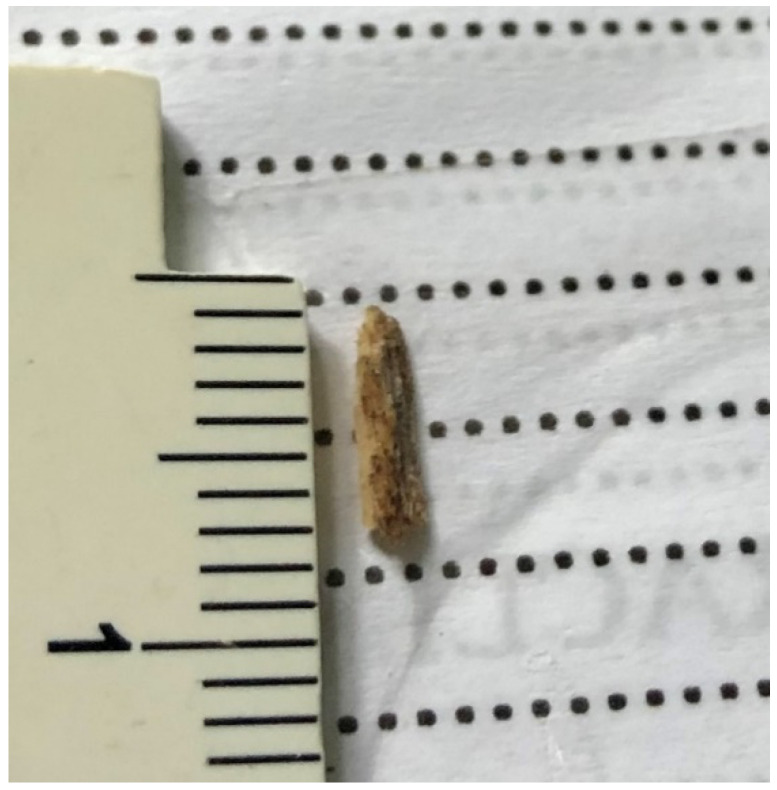
Large IOFB (7 mm). IOFB=Intraocular foreign body.

The average IOFB length was 3.54 mm, ranging between 0.5 mm and 8 mm (Figure 1 and Figure 2). According to ocular trauma classification [21], the entry site of the IOFB was in zone 1 in 38 cases (67.8%), zone 2 in 7 cases (12.5%) and zone 3 in 11 cases (19.7%). In 29 cases, the IOFB was located in the vitreous cavity (51.8%) and in 27 cases, it was embedded in the retina (48.2%). Most of the IOFBs were metallic (54 cases—96.4%) and two (3.6%) were made of wood; the size was ≤3 mm in 26 cases (46.4%) and >3 mm in 30 cases (53.6%). We correlated all the abovementioned parameters with the final BCVA and statistical significance was obtained only when regarding the location: retinal IOFBs had a significantly worse functional outcome as compared to the intravitreal ones (*p* = 0.046). 

### 3.3. Initial BCVA, Visual Outcome and Prognostic Factors

**Figure 3:**
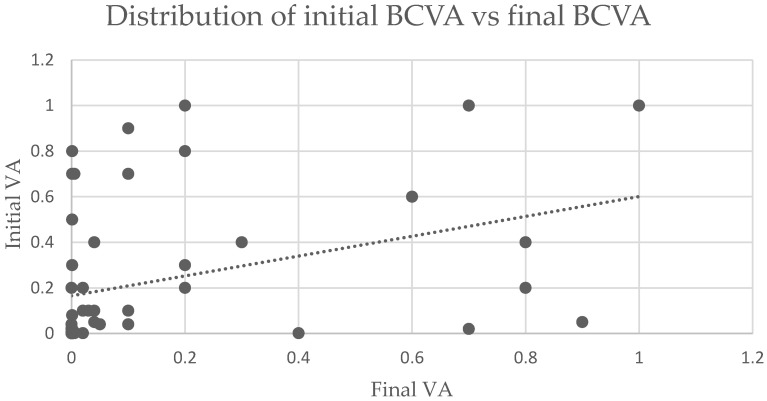
Distribution of initial BCVA vs. final BCVA. BCVA= Best corrected visual acuity.

The preoperative and postoperative BCVAs are listed in Figure 3. Final BCVA was declared as the VA noted at the last follow-up. The length of follow-up varied between 1 and 24 months, with a mean of 3.4 months. In order to outline the prognostic factors, we defined the outcome according to the value of final BCVA as follows: poor (final BCVA <0.1), good (final BCVA 0.1–<0.5) and excellent (final BCVA ≥0.5). Among the study group, 38 (67.9%) patients had poor BCVA at presentation, while this proportion significantly decreased to 53.6% following surgery. The number of cases with good BCVA at presentation vs. after surgery increased from 11 (19.6%) to 15 (26.8%), respectively. The number of cases with excellent BCVA after surgery increased from 7 cases (12.5%) preoperatively to 11 cases (19.6%) postoperatively. 

Univariate analyses of the factors associated with final BCVA are summarized in Table 2. The results indicate that initial VA (*p* < 0.018), the retinal location of the IOFB (*p* < 0.046), the association of RD at initial examination (*p* < 0.010) and endophthalmitis (*p* < 0.040) were risk factors associated with a poor visual outcome. 

From the 27 cases with retinal IOFB, only 8 (29.6%) recovered useful vision (BCVA ≥ 0.1), whereas from the 29 cases with intravitreal IOFB, 18 (62.1%) ended up with a good visual outcome (BCVA ≥ 0.1) (*p* = 0.046). 

RD was identified in 24 patients (42.85%), of which 12 occurred at the time of presentation and 12 during follow-up. Among the 12 patients with RD at presentation, only 1 (8.0%) achieved final BCVA ≥0.1, whereas from the 44 cases without RD at presentation, 24 (54.6%) recovered final BCVA ≥0.1 (*p* = 0.010). Within the group of 12 patients with RD during follow-up, 5 cases had endophthalmitis upon presentation and in 1 case, the foreign body was in the orbit; 11 patients had final BCVA of CF or less and only 1 maintained BCVA ≥ 0.1. 

Endophthalmitis was associated in 17 cases (30.4%) and compromised the visual outcome in 13 of them (76.5%). Only four patients (23.5%) within the endophthalmitis group reached final BCVA ≥0.1. Among the patients without endophthalmitis (39 cases—69.6%), 22 (56.4%) had final BCVA ≥0.1 (*p* = 0.040). 

In our study, the age, IOFB size, entry site time of extraction and lens injury did not have a statistically significant impact on the final BCVA (Table 2). The time interval between trauma and IOFB removal ranged from 1 to 30 days, with a mean of 3.3 days. Of all 56 patients, 30 (53.6%) underwent PPV within the first 48 h, 20 (35.7%) within 7 days and 6 (10.7%) after 1 week. Although it did not reach statistical significance (*p* = 0.208), PPV that was performed later carried out a higher risk for poor visual outcome. From the 26 cases in which PPV was performed later than 48 h from injury, 17 (65%) had a final BCVA <0.1. Of the 30 cases where PPV was performed within the first 48 h, 17 (57%) had a final BCVA ≥0.1. 

The surgical treatment is summarized in Table 3. The length of hospitalization for the first hospital admission was 8.16 ± 5.61 days (range 2–29). The case that required the longest hospitalization, 29 days, was a trauma with explosive material that caused extensive eye injuries, corneal impregnation with foreign bodies, marked corneal oedema that made PPV impossible in early stages and also a complex left-hand lesion that required a skin graft. All patients underwent PPV within the first 24 h from admission combined with primary repair of the globe in 25 cases. Because our hospital is a tertiary care unit, the repair of the primary lesion was performed in the referring facility in six cases. IOFB was extracted in 53 of the 56 eyes included in the study (94.6%), whereas in three cases, IOFB extraction failed due to its intraorbital location.

The other primary surgical procedures were lensectomy in 25 cases (44.6%), phacoemulsification in 3 cases (5.4%), suture of the globe in 25 cases (44.6%), intravitreal antibiotic injection in 12 cases (21.4%) and laser of the retinal lesion in 24 cases (42.9%). Subsequent surgeries were performed as follows: PPV 21 cases (37.50%), phacoemulsification 5 cases (8.9%), IOL implantation 12 cases (21.4%) and intravitreal antibiotic injection 5 cases (8.9%). 

Of the 28 eyes that underwent lensectomy or phacoemulsification as part of the first intervention, in 4 of them, IOL was implanted at the end of surgery, and in 12, IOL was planted at a later stage; 12 eyes remained aphakic. Ten of the cases that remained aphakic exhibited extensive ocular damage with low potential for visual improvement. The other two cases presented concurrent endophthalmitis and we made the decision to have a secondary IOL implantation at a later stage but, for unknown reasons, these 2 patients were lost during follow-up. For eight eyes, lensectomy or phacoemulsification was performed at a later stage and was followed by IOL implantation in three eyes. Overall, 17 eyes remained aphakic. 

Endolaser photocoagulation of the retinal lesion produced by the IOFB was carried out in 24 eyes, of which 6 developed RD at a later stage. 

Overall, 24 eyes were treated for RD. Of these, 12 (50%) were present at the initial setting, and 12 occurred later (50%); 10 had concurrent endophthalmitis and 3 cases were seen with intraorbital foreign body. In all cases in which visibility was permitted, reattachment of the retina was performed and silicone oil was used for internal tamponade. Among all patients with RD, the final BCVA was ≤ hand motion in 14 cases, CF in 8 cases and >0.1 in 2 cases. Proliferative vitreoretinopathy (PVR) was noted in 9 out of 24 eyes with RD (37.5%). Among the nine PVR cases, eight cases had IOFB ≥ 3mm, four cases had concurrent endophthalmitis and four cases underwent PPV after 48 h. 

We also analyzed the risk factors for RD, which are summarized in Table 4. The univariate analysis evaluated age, concurrent endophthalmitis, IOFB location and size, as well as the entry site. In the whole study group, the IOFB was embedded in the retina in 27 cases; of these, 18 cases presented with RD (66.7%). Within the intravitreal IOFBs group (29 cases), only six patients developed RD (20.68%). This difference is statistically significant (*p* < 0.001). We also found that the IOFBs larger than >3 mm carried a higher risk for RD compared to smaller ones: of the 30 eyes with IOFB >3 mm, 17 (56.6%) developed RD, whereas of the 26 cases with IOFB ≤ 3mm, 7 (26.9%) developed RD (*p* = 0.02). In the postoperative RD subgroup, the IOFB size was not correlated with a higher risk of RD (*p* = 0.29). Endophthalmitis did not represent an indicator for RD development in any subgroup, but it did show an increased predisposition to RD in the whole group; of the 17 cases with endophthalmitis, 10 (58.8%) developed RD, and of the 39 eyes without endophthalmitis, 14 developed RD (35.9%) (*p* = 0.11).

Post-traumatic endophthalmitis was present in 17 eyes, 12 eyes received intravitreal antibiotic injections with vancomycin 1.0 mg/0.1 cc and/or ceftazidime 2.2 mg/0.1 cc at the time of primary repair and for 5 eyes, intravitreal therapy was given at 1 day (one case), 2 days (one case), 3 days (one case), 4 days (one case) and 37 days (one case) after the primary repair. In all cases, systemic moxifloxacin was administered (400 mg/day). Regarding the final BCVA, excellent outcome was seen in 3 cases (17.6%), good outcome in 1 case (5.9%) and poor outcome in 13 cases (76.5%).

The indications for undergoing additional PPV were: RD (14 cases), endophthalmitis (3 cases), epiretinal membrane (1 case), cataract (1 case), combined RD and cataract (1 case), combined endophthalmitis, RD and cataract (1 case) and retinal break (1 case). The average number of surgical procedures performed per patient was 2.14 ± 1.25 (range, 1–8).

## 4. Discussion

Eye trauma is an important and preventable cause of unilateral blindness, with penetrating eye injuries taking first place [2]. In this context, retained IOFBs reveal complex clinical scenarios that can lead to the compromise of vision. According to the World Health Organization (WHO), there are 1.9 million persons with monocular blindness or low vision due to ocular trauma [2]. Vision loss can be of different extents, depending on complications, location and size of the injury. Most of these patients are young, healthy, working-age men with a long life expectancy and for whom quality of life is (QoL) a major issue [1,6,22]. In addition to the negative impact that vision impairment has on mental health, individuals face other problems related to social stigma, inability to maintain a certain job and support their families and themselves. Many patients must either reorient themselves professionally, or obtain a certificate of disability, which has a significant negative impact on their QoL [23]. A study conducted by Yuksel et al. assessed the impact of penetrating ocular injuries on the quality of life and psychological status among patients with penetrating ocular trauma and demonstrated that they have increased psychological symptoms and poor QoL compared to healthy subjects [24]. Schrader et al. also reported a decrease in QoL and economic condition, particularly in cases with retinal involvement, and mentioned that some patients were forced to discontinue social activities and hobbies such as motorcycling, squash, soccer or driving [25]. 

In this retrospective study, we analyzed the data of 56 patients with the aim to outline the prognostic factors and complications associated with final BCVA in penetrating ocular injuries with retained IOFB during an 11-year period. The average age of our patients was 36.1 ± 14.1 years and included a total of 55 males (98.2%), which is similar to the demographic characteristics of other reports [1,6,22,26]. Young men are more exposed to eye injuries due to the fact that they carry out more risky activities in this regard. The most common factors associated with the production of eye injuries were not wearing goggles and metal hammering [1,6,9,14,15,16,18,19,20,22,27,28]. In our case series, none of the subjects wore goggles, which is why we strongly support their crucial role in preventing eye damage. The main reason why our cohort did not wear eye protection is the lack of proper education. Furthermore, most of the accidents happened during household activities and not at the workplace, where safety regulation requires employers to provide eye protection in at-risk occupations. 

Several authors [7,22] found that patients who are older than 50 years have poorer visual outcome when compared to younger ones; however, in our study, age had no impact on final BCVA (*p* = 0.270). The explanation for this observation lies in the fact that most of the patients in our series are young, due to the different mechanism by which eye trauma occurs in the elderly compared to the young. More precisely, eye trauma in the geriatric population most often results in globe ruptures due to falls and rarely involves an IOFB, which is in contrast with young males with an occupational-related open globe injury and IOFBs [29]. 

Initial poor BCVA was correlated with a poor prognosis throughout our study (*p* = 0.018). Previous research indicated that poor BCVA at presentation is a major risk factor for poor visual outcome, mainly because it reflects a higher degree of ocular damage at presentation [13,14,15,22,26]. In contrast, some authors did not find initial BCVA to be correlated with a poor visual prognosis, mentioning that vitreous hemorrhage or traumatic cataract, which can significantly affect initial BCVA, can be successfully resolved by surgery without impacting final BCVA [9,20,27]. In our study, timing of surgery did not correlate with final visual outcome (*p* = 0.208), but a delay in surgery of more than 48 h showed a higher risk for poor visual outcome with 17 cases of 26 (56.7%) ending up with final BCVA <0.1. Timing of surgery (delayed vs. immediate) is a well-discussed, controversial topic and depends on several factors such as patient’s general health condition, presence or absence of endophthalmitis and the availability of well-trained surgeons and personnel. On the one hand, there are authors who pointed out that late PPV and IOFB removal (after 24 h) increases the risk of developing endophthalmitis and PVR [3,6,30,31] and recommended PPV within the first 24 h from the accident. On the other hand, several studies found no additional risk regarding endophthalmitis in late PPV and IOFB removal, with a delay of 1 day to 3 years in diagnosis and treatment, suggesting that other prognostic factors such as initial BCVA, RD and lens injury play important roles for the final visual outcome [16,32]. Early PPV offers some advantages, such as toxins and inflammatory cells clearance, restoration of ocular media clarity, allowing specimen cultures, providing better diffusion of intravitreal antibiotics and eliminating the vitreous scaffold which favors retinal traction. However, surgery on a severely traumatized eye is accompanied by the risk of iatrogenic complications due to poor visualization of the posterior segment caused by anterior segment lesions or vitreous hemorrhage. Delaying IOFB extraction may result in the resolution of corneal edema and/or hyphema and better wound integrity. Against the popular conception that delaying PPV will possibly lead to spontaneous posterior vitreous detachment (PVD), Kuhn named this as one of the most misleading statements in VR surgery and that true PVD among young individuals does not occur; therefore, this argument should not be taken into consideration [33].

Regarding the size of IOFBs, some authors indicated that IOFBs ≥3mm are an important independent risk factor for poor visual outcome, suggesting that the larger the IOFB, the greater the kinetic energy that develops when it penetrates the eye, which increases the risk of retinal damage [1,9,16,17,18,19,34]. On the other hand, one work of research found that the size of the IOFB had no impact on the visual function [14]. The explanation lies in the fact that the edge of the foreign body that penetrates the eye and the surface of the retina is not necessarily the largest size of the foreign body. Unlike our previous results, in this study, the final BCVA was not significantly correlated with the size of the IOFB (*p* = 0.105) [1].

The location of the entry site did not prove to be a prognostic factor for the visual outcome in our series (*p* = 0.932), which corresponds to previous findings [15,28]. This finding may be related to the small number of participants and the unequal distribution, with corneal lesion comprising 67.8%. Additionally, there were other associated prognostic factors such as post-traumatic corneal oedema and inflammation interfering with intraoperative visualization and the late referral at our tertiary care center with subsequent increased risk of inflammation and endophthalmitis. Of all cases of endophthalmitis with poor visual outcome (13 cases), zone I lesion represented 53.8% (7 cases). Unlike zone of injury, significant difference was found between the intravitreal and retinal location of the IOFB, with the latter having a significantly poorer visual outcome (*p* = 0.046). It is well-documented in the literature that posterior segment IOFBs are more likely to damage the retina and cause RD and ir-reversible vision loss [6,16,19,35]. Contrary to our findings, Anguita et al. observed that IOFBs in the posterior segment were not associated with poor visual outcome, assuming that the energy of the IOFB had been absorbed by the anterior segment (cornea, iris, lens) and the IOFB landed in the vitreous cavity, sparing the retina [28]. 

Post-traumatic cataract is a common complication of open globe injuries with IOFB, ranging from 44% to 66% of cases [36]. Traumatic cataract can develop immediately after the eye injury or weeks or months later. Despite the initial changes in visual acuity due to the lens opacification, if the integrity of the retina (especially of the macula) is preserved, visual acuity could be improved after surgery [27]. Lens injury was not associated with poorer visual outcome in our series (*p* = 0.974). 

The primary cause of vision loss in penetrating ocular injuries with posterior segment IOFB is the development of RD and PVR. Preoperative and postoperative RD remain a frequent complication associated with high risk of vision-threatening consequences despite all surgical progress made in ocular trauma management [1,8,15,20,26]. In our case series, RD was one of the independent risk factors for poor visual outcome. From all the 25 cases with RD included in this study, only 2 had final BCVA ≥0.1. Furthermore, we analyzed the potential risk factors involved in the development of RD (Table 4). We found that IOFBs larger than 3 mm and located intraretinally strongly predicted the development of RD, which is consistent with previous findings [16]. Several authors also described scleral/corneoscleral entry [16] and endophthalmitis [8] to be important predictive factors for the development of postoperative RD. However, our results did not show a correlation between the development of postoperative RD and IOFB size, entry site and endophthalmitis. In a recent study, Brodowska et al. published the validation of RD after Open Globe Injury Score (RD-OGI SCORE), which can predict the future risk of RD based on clinical findings at initial presentation [37].

PVR following open globe injuries is much more frequent than in primary RD, occurring in 40–60% of patients [38]. The high incidence of PVR after ocular trauma is thought to be due to disruption of the blood–retinal barrier that promotes the intraocular inflammatory response. As a result, proliferation of fibroblasts and glial cells occurs, leading to epiretinal and subretinal membrane formation and subsequent traction. The fibrocellular proliferation response can develop within days after injury, causing permanent damage to the retina [39]. In terms of PVR management and development, Assi et al. [40] described in their study the potential role of early intraocular Mitomycin C applications at the site of chorioretinal injury in reducing the rate of proliferation, and another study showed that Methotrexate [41] used on PVR cells obtained in vitro can significantly reduce growth and induce cell death. A surgical option is chorioretinectomy, which consists of endodiathermy around the wound to destroy the retina and choroid and create a barrier between the scar and remaining retinal edge. It was first described by Kuhn and coworkers [42] and mentioned in several studies proving the decreased rate of PVR and better visual outcomes [43,44]. In our study, PVR was noted in 9 (37.5%) of the 24 eyes with RD. 

Traumatic endophthalmitis is a devastating complication of open globe injuries, with an incidence ranging from 0% to 17% in different studies [9,10,11,12]. The prevalence of endophthalmitis in our series was 30.4%, which is higher than in other studies [11,12,35]. Although all endophthalmitis patients underwent PPV within the first 24 h after admission, the median time to IOFB removal was 3 days (range 1–8). We attribute this finding to the late referral of the patients to our center. Another possible explanation of the high incidence of endophthalmitis in our series is the absence of primary wound closure, with only one case within the endophthalmitis subgroup in which suture had been performed prior to IOFB removal. Despite the delays in IOFB extraction due to late presentation, with 11 out of 17 cases of endophthalmitis in which IOFB was extracted more than >48 h after injury (64.7%), no impact on final visual outcome was observed (*p* = 0.376). Immediate PPV with IOFB removal has been described to reduce the incidence of endophthalmitis [3,6,16,30,31]. However, Colyer et al. demonstrated that timing of IOFB removal did not influence the risk of endophthalmitis among military members injured during war and pointed out the efficacy of topical and systemic fluoroquinolone agents (gatifloxacin, moxifloxacin or levofloxacin) in reducing the risk of infection [11]. Nevertheless, we support the idea that in all cases of ocular trauma with IOFB complicated with endophthalmitis, surgery must be done as soon as possible. The IOFBs described in the abovementioned study were represented by shrapnel that sterilize in the air due to their very high temperature and speed [11].

This study has some limitations, represented by its retrospective nature, with all data being collected from medical charts; the relatively small number of patients; and the lack of long-term information regarding the patients’ visual outcome.

## 5. Conclusions

Poor visual outcome after posterior segment IOFBs is strongly related to initial BCVA, RD at presentation, endophthalmitis and retinal location of the IOFB. The retinal location of the IOFB is significantly associated with the occurrence of RD. Because the IOFBs represent a cause of potential visual morbidity and blindness among young, working-class men, eye protection during prone activities is crucial and needs more promotion and education.

## Figures and Tables

**Table 1:** Demographic data of patients.

Variable	BCVA < 0.1	BCVA 0.1–<0.5	BCVA ≥ 0.5	*p* Value
Age	35.5 (25.5; 47)	39 (23; 50)	32 (27; 43)	0.809
Gender				
Female	1 (3.3%)	0 (0%)	0 (0%)	0.643
Male	29 (96.7%)	15 (100%)	11 (100%)	
Eye				
Right	12 (40%)	7 (46.6%)	5 (45.5%)	0.697
Left	18 (60%)	8 (53.3%)	6 (54.5%)	
Eye protection				
Yes	-	-	-	
No	30 (100%)	15 (100%)	11 (100%)	
Location				
Rural	24 (80.0%)	10 (66.7%)	6 (54.5%)	0.249
Urban	6 (20%)	5 (33.3%)	5 (45.5%)	
Type of accident				
Work	2 (6.7%)	1 (6.7%)	1 (9.1%)	0.962
Daily life	28 (93.3%)	14 (93.3%)	10 (90.9%)	

BCVA: Best corrected visual acuity.

**Table 2:** Factors influencing final BCVA (univariate analysis of factors affecting the final visual outcome of patients with IOFB).

Factor	Nr. Eyes	BCVA < 0.1	BCVA 0.1- < 0.5	BCVA ≥ 0.5	*p*-Value
Age					
≤50 years	47 (83.9%)	24 (80%)	12 (80%)	11 (100%)	0.270
>50 years	9 (16.1%)	6 (20%)	3 (20%)	0 (0%)	
IOFB location					
Retina	27 (48.2%)	19 (66.3%)	4 (26.7%)	4 (36.4%)	**0.046**
Vitreous	29 (51.8%)	11 (36.7%)	11 (73.3%)	7 (63.6%)	
Entry site					
Cornea	38 (67.8%)	29 (63.3%)	11 (73.4%)	8 (72.7%)	0.932
Sclera <5 mm	7 (12.5%)	4 (13.3%)	2 (13.3%)	1 (9.1%)	
Sclera >5 mm	11 (19.7%)	7 (23.4%)	2 (13.3%)	2 (18.2%)	
Endophthalmitis					
Yes	17 (30.4%)	13 (43.3%)	1 (6.7%)	3 (27.3%)	**0.040**
No	39 (69.6%)	17 (56.7%)	14 (93.3%)	8 (72.7%)	
RD at presentation					
Yes	12 (21.4%)	11 (36.6%)	1 (6.7%)	0 (0%)	**0.010**
No	44 (78.6%)	19 (63.3%)	14 (93.3%)	11 (100%)	
Lens Injury					
Yes	36 (64.3%)	19 (63.3%)	10 (66.7%)	7 (63.6%)	0.974
No	20 (35.7%)	11 (36.7%)	5 (33.3%)	4 (36.4%)	
Initial VA					
<0.1	38 (67.9%)	26 (86.7%)	8 (53.3%)	4 (36.4%)	**0.018**
0.1–<0.5	11 (19.6%)	2 (6.7%)	5 (33.3%)	4 (36.4%)	
≥0.5	7 (12.5%)	2 (6.7%)	2 (13.3%)	3 (27.3%)	
IOFB size					
≤3 mm	26 (46.4%)	10 (33.3%)	9 (60%)	7 (63.6%)	0.105
>3 mm	30 (53.6%)	20 (66.7%)	6 (40%)	4 (36.3%)	
IOFB type					
Metallic	54 (96.4%)	29 (96.7%)	14 (93.3%)	11 (100%)	0.660
Nonmetallic	2 (3.6%)	1 (3.3%)	1 (6.7%)	0 (0%)	
IOFB time extraction					
<48 h	30 (53.6%)	13 (43.3%)	9 (60%)	8 (72.7%)	0.208
>48 h	26 (46.4%)	17 (56.7%)	6 (40%)	3 (27.3%)	

Nr = Number. BCVA = Best corrected visual acuity. IOFB = Intraocular foreign body. RD = Retinal detachment. VA = Visual acuity. Bold: *p* value was significant.

**Table 3:** Surgical treatment.

Variable	Number of Eyes with IOFB
IOFB initially retrieved	53 (94.6%)
Number of hospitalization days 1st admission	8.16 ± 5.61
Primary surgical procedures performed	
PPV with IOFB extraction	53 (94.6%)
Lensectomy	25 (44.6%)
Phacoemulsification	3 (5.4%)
Suture of wound	25 (44.6%)
Intravitreal antibiotic injection	12 (21.4%)
Laser of retinal impact site	24 (42.9%)
Additional surgical procedures performed	
PPV	21 (37.5%)
Phacoemulsification	5 (8.9%)
IOL implantation	12 (21.4%)
Intravitreal antibiotic injection	5 (8.9%)
Number of surgical procedures performed	2.14 ± 1.25

IOFB = Intraocular foreign body. PPV = Pars plana vitrectomy. IOL = Intraocular lens.

**Table 4:** Factors influencing RD development.

RD—Overall				RD—Postoperative			
Factor	Yes	No	*p* Value	Factor	Yes	No	*p* Value
Age				Age			
≤50 years (*n* = 47)	19(39.6%)	28(60.4%)	0.40	≤50 years (*n* = 37)	9(24.3%)	28 (75.7%)	0.34
>50 years (*n* = 9)	5 (55.6%)	4 (44.4%)		>50 years (*n* = 7)	3 (42.9%)	4 (57.1%)	
Endophthalmitis				Endophthalmitis			
Yes (*n* = 17)	10(58.8%)	7 (41.2%)	0.11	Yes (*n* = 12)	5 (41.7%)	7 (58.3%)	0.18
No (*n* = 39)	14(35.9%)	25(64.1%)		No (*n* = 32)	7 (21.9%)	25 (78.1%)	
IOFB size				IOFB size			
≤3 mm (*n* = 26)	7 (26.9%)	19(73.1%)	**0.02**	≤3 mm (*n* = 24)	5 (20.8%)	19 (79.2%)	0.29
>3 mm (*n* = 30)	17(56.7%)	13(43.3%)		>3 mm (*n* = 20)	7 (35.0%)	13 (65.0%)	
IOFB location				IOFB location			
Retina (*n* = 27)	18(66.7%)	9 (33.3%)	**0.000**	Retina (*n* = 18)	9(50%)	9 (50%)	**0.004**
Vitreous (*n* = 29)	6 (20.7%)	23(79.3%)		Vitreous (*n* = 26)	3 (11.5%)	23 (88.5%)	
IOFB entry site				IOFB entry site			
Zone I (*n* = 38)	14(35.9%)	24 (64.1%)	0.28	Zone I (*n* = 32)	8 (25%)	24 (75%)	0.85
Zone II (*n* = 7)	3 (42.9%)	4 (57.1%)		Zone II (*n* = 6)	2 (33.3%)	4 (66.7%)	
Zone III (*n* = 11)	7 (63.6%)	4 (36.4%)		Zone III (*n* = 6)	2 (33.3%)	4 (66.7%)	

RD = Retinal detachment. IOFB = Intraocular foreign body. Bold: *p* value was significant.

## Data Availability

The data presented in this study are available on request to the corresponding author.
